# Steroid-Resistant Double-Seronegative Optic Neuritis Responds Favorably to Plasma Exchange

**DOI:** 10.7759/cureus.15260

**Published:** 2021-05-26

**Authors:** Matthew C Mason, Dario A Marotta, Hassan Kesserwani

**Affiliations:** 1 Department of Research, Alabama College of Osteopathic Medicine, Dothan, USA; 2 Department of Neurology, Division of Neuropsychology, University of Alabama, Birmingham, USA; 3 Department of Neurology, Flowers Medical Group, Dothan, USA

**Keywords:** bilateral optic neuritis, double seronegative optic neuritis, plasma exchange therapy, neuromyelitis optica spectrum disorder, myelin-oligodendrocyte glycoprotein (mog), steroid resistant optic neuritis

## Abstract

The clinical presentation of optic neuritis is quite characteristic, and the epidemiology, differential diagnosis, and treatment protocol are well established. However, when the presentation of optic neuritis is atypical, bilateral, and intravenous steroid-resistant, the treatment guidelines are quite nebulous. We present a case of bilateral severe double-seronegative optic neuritis with catastrophic vision loss and intravenous steroid resistance. After an exhaustive investigation, we empirically treated our patient with plasma exchange therapy and obtained a dramatic recovery of vision. When an immune etiology is suspected, this case is instructive vis-a-vis the utility of plasma exchange in refractory cases of optic neuritis despite seronegativity.

## Introduction

Optic neuritis is usually due to inflammatory demyelination of the optic nerve that typically presents with acute pain, monocular vision loss [1,2]. The incidence of optic neuritis is approximately 1.5-5.1 cases per 100,000 with a strong predilection to females [3,4]. It commonly occurs in multiple sclerosis (MS), however, there are other causes that include infectious, ischemic, autoimmune, and hereditary etiologies [5]. While originally thought to be related to MS, neuromyelitis optica (NMO) and myelin oligodendrocyte glycoprotein antibody disease (MOGAD) have distinct differences in clinical presentation, serological markers, imaging findings, response to treatment, and clinical outcomes [6-8]. One key component of this distinction was the identification of the MOG autoantibody for MOGAD and the aquaporin-4 (AQP-4) autoantibody for NMO [9,10]. One of the most common presenting symptoms of these syndromes is optic neuritis. However, NMO and MOGAD, when compared with MS, typically present with more severe acute onset vision loss that is either unilateral, sequential, or simultaneously bilateral [11,12]. Here, we report a 35-year-old female with acute onset and sequential vision loss with seronegativity to both AQP-4 and MOG, and no obvious etiology after a comprehensive evaluation, who failed to respond to intravenous methylprednisolone (IVMP) but responded robustly to plasma exchange (PLEX) therapy.

## Case presentation

A 35-year-old African American female presented to the neurology clinic with vision loss in the right eye. The patient reported that she was evaluated at the emergency department (ED) nine days prior to sudden onset painful vision loss in her right eye with associated transient visual obscurations, pulsatile tinnitus, headache, and nausea. At that time, she received magnetic resonance imaging (MRI) of the brain with and without gadolinium, which was normal. She was also empirically treated with three days of daily one-gram IVMP without improvement.

In the nine days following her evaluation at the ED and before arriving in the clinic, her vision gradually worsened to the point she was unable to read. She now required guidance to navigate her surroundings. She denied any numbness, balance issues, diplopia, fatigue, urogenital dysfunction, dizziness, or slurred speech. She also denied cough, shortness of breath, rashes, and arthralgias. The patient reported a history of second-trimester pregnancy loss, but otherwise, the patient reported a non-significant past medical history with sporadic routine care from her primary care physician. She denied a history of tobacco, alcohol, and illicit drug use. Family history was also unremarkable and without any history of thrombosis or other underlying autoimmune diseases.

On physical examination, the patient was 5 feet 4 inches, weighed 212 pounds with a body mass index of 36.4. The patient was ambulating without difficulty and had a normal gait and posture. Romberg sign was absent. Facial strength was full with no asymmetry. The gross hearing was intact. Her right eye could barely perceive light. She was definitely unable to count fingers. Her left eye had some light perception with a vague perception of images, which she described as shadows. Both eyes exhibited a Marcus-Gunn pupil. Accommodation could not be tested due to poor visual acuity. An extra-ocular motion was full in all directions. The rest of the cranial nerves were normal. Muscle strength testing in all extremities was 5/5 on the medical research council grading for muscle strength. Deep tendon reflexes were 2+ bilaterally in the upper and lower extremities. Sensory examination to touch-pressure, pin-prick, and joint-position sense was normal in the fingers and toes.

Lumbar puncture revealed a normal cerebral spinal fluid (CSF) pressure of 15 cm of water. CSF analysis and plasma serology are displayed in Table 1.

**Table 1:** Results of CSF and serological analyses CSF = Cerebral spinal fluid; N/A = Not applicable; RBC = Red blood cell; Ig = Immunoglobulin; ALB = albumin; NMO = Neuromyelitis optica; MOG = Myelin oligodendrocyte glycoprotein; ANA = Antinuclear antibody; RPR = Rapid plasma reagin; INR = International normalized ratio; APTT = Activated partial thromboplastin time; DRVVT = Dilute Russell's viper venom time

TEST	RESULT	INTERPRETATION	REFERENCE RANGE	UNITS
CSF				
Color	Colorless	Normal	Colorless	N/A
Clarity	Clear	Normal	Clear	N/A
Total protein	14.0	Normal	0.0-44.0	mg/dL
CSF glucose	58	Normal	40-70	mg/dL
Nucleated cell	0	Normal	0-5	cells/
RBC	0	Normal	None seen	cells/
IgG, quantitative	1.5	Normal	0.0-8.6	mg/dL
Albumin	9	Low	11-48	mg/dL
IgG/ALB ratio	0.17	Normal	0.00-0.25	Ratio
IgG Index	0.4	Normal	0.0-0.7	Ratio
Oligoclonal bands	0	Normal	<4	N/A
SERUM				
NMO IgG autoantibodies	<1.5	Normal	0.0-3.0	U/mL
MOG autoantibodies	Negative	Normal	Negative	N/A
ANA	Negative	Normal	Negative	N/A
RPR	Non-reactive	Normal	Non-reactive	N/A
IgG	1,518	Normal	586-1,602	mg/dL
Albumin	4.0	Normal	3.8-4.8	g/dL
Prothrombin time	10.5	Normal	9.1-12	Seconds
INR	1.0	Normal	0.9-1.2	Ratio
APTT	23.0	Normal	22.9-30.2	Seconds
Thrombin time	17.2	Normal	0.0-23.0	Seconds
DRVVT screen seconds	31.1	Normal	<47.0	Seconds
Hexagonal phospholipid neutral	0	Normal	0-11	Seconds
Platelet neutralization	0.0	Normal	0.0-3.0	Seconds
Anticardiolipin IgG	<10	Normal	<15	G phospholipids
Anticardiolipin IgM	<10	Normal	<21	M phospholipids
Beta-2 glycoprotein IgG	<10	Normal	<21	Standard IgG units
Beta-2 glycoprotein IgM	<10	Normal	<33	Standard IgM units
Beta-2 glycoprotein IgA	<10	Normal	<26	Standard IgA inits
Lupus anticoagulant	Not detected	Normal	Not detected	Standard assessment units

Specifically, MOG autoantibodies and AQP-4 autoantibodies were negative, a condition termed double-negative serology. CSF analysis was non-inflammatory with absent bands and normal immunoglobulin indices. Chest computerized axial tomography (CT) scan revealed no hilar adenopathy, and carotid duplex ultrasound of the carotid arteries showed no stenosis. An additional course of five days of daily one-gram IVMP again did not improve her vision.

The patient returned to the clinic five weeks later with new onset painful blurry vision in her left eye associated with fatigue, myalgias, diffuse weakness, and back pain. However, she reported no change in her right eye visual acuity. Physical examination revealed the right eye to be light perceptive but without the ability to make out images. She was also unable to count fingers with the left eye, but she had better light perception. A repeat MRI of the brain and orbits with and without contrast revealed enhancement of the right optic nerve and its sheath in its intracanalicular and prechiasmatic portion. There was also evidence of enhancement in the left optic nerve.

**Figure 1:**
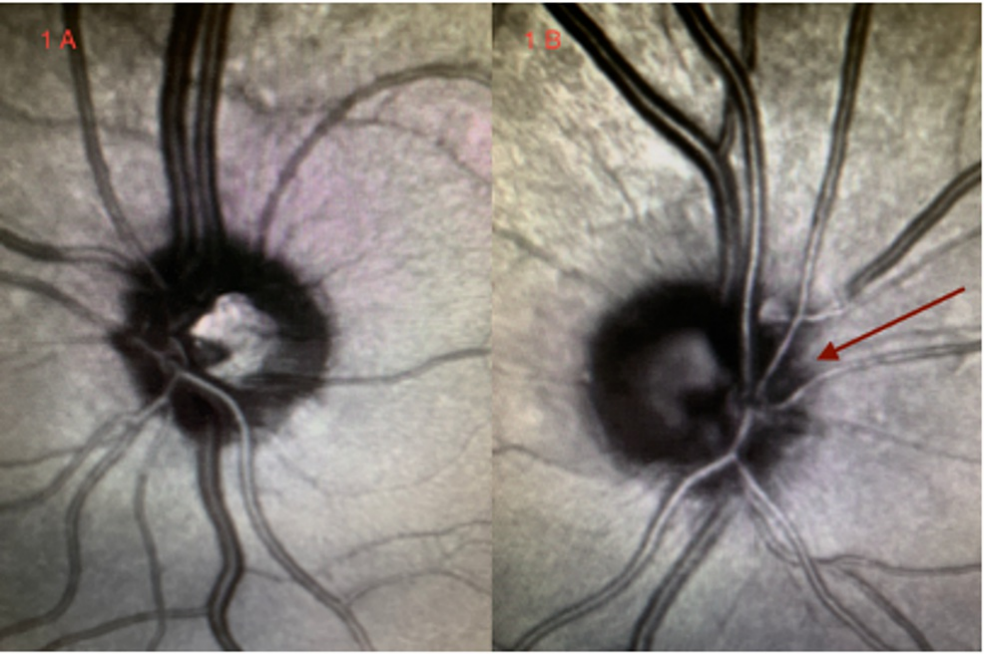
Funduscopic eye examination. (A) Left eye: normal optic disc. (B) Right eye: nasal optic disc pallor (red arrow).

**Figure 2:**
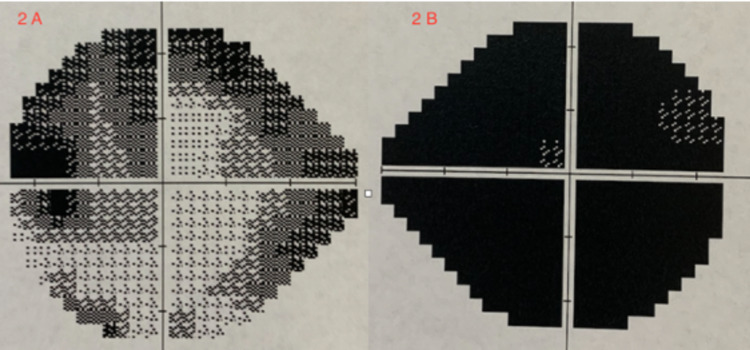
Humphrey visual field testing. (A) Left eye: severe visual field constriction. (B) Right eye: black-out of visual field test.

A neuro-ophthalmological evaluation revealed pupils that were equal, round, reactive, and with bilateral afferent pupillary defect (Marcus-Gunn pupil). The cornea, lens, iris, and anterior chamber were normal. The maculae, retinal vessels, vitreous humor, and left optic nerve were normal. The right optic nerve showed mild nasal atrophy. Fundal photos showed mild nasal pallor in the right eye and normal in the left (Figures 1, 2).

Optical coherence tomography (OCT) revealed normal thickness of the retinal nerve fiber layer (RNFL) and ganglion cell layers (GCL) bilaterally. Ishihara color test showed 0/8 and 6/8 in the right eye and left eye, respectively. Humphrey visual field testing of the right eye showed black-out of the visual field, while the left visual field showed severe constriction (Figures 2A, 2B).

Due to the catastrophic vision loss and steroid resistance, PLEX therapy was delivered at a total dose of 250 mL per kg, with a total of six PLEXs, delivered every other day. The patient reported a dramatic improvement in her visual acuity over the course of two weeks and she was able to watch television and ambulate without assistance. The formal ophthalmologic evaluation revealed resolution of the Marcus-Gunn pupil with refractive correction of visual acuity to 20/40 bilaterally.

## Discussion

In the United Kingdom 22-year cohort analysis of 2,826 patients, the incidence rate of optic neuritis was 3.7 per 100,000 patient-years. This study showed a female predominance of 92.4% and an incidence rate that showed minimal fluctuation over the two decades. By far, MS had the strongest association with optic neuritis, followed by Behcet's disease, sarcoidosis, ocular syphilis, and vasculitis, in descending order of frequency, which is seen in Table 2 [13].

**Table 2:** Odds ratios and hazard ratios for conditions associated with optic neuritis CI = Confidence interval

CONDITION	ODDS RATIO (PRIOR); 95% CI	HAZARD RATIO (INCIDENT); 95% CI
Multiple Sclerosis	98.22 (65.40-147.52)	284.97 (167.85-483.81)
Syphilis	5.76 (1.39-23.96)	
Mycoplasma	3.90 (1.09-13.93)	
Vasculitis	3.70 (1.68-8.15)	4.89 (1.82-13.10)
Sarcoidosis	2.50 (1.21-5.18)	14.80 (4.86-45.08)
Epstein-Barr	2.29 (1.80-2.92)	
Crohn’s Disease	1.97 (1.13-3.43)	
Psoriasis	1.28 (1.03-1.58)	
Sjogren Syndrome		3.48 (1.38-8.76)
Herpes Infection		1.68 (1.24-2.28
Behcet’s Disease		17.39 (1.55-195.53)

In the Japanese epidemiologic study of 531 cases of optic neuritis, the authors noted that 77% of patients with optic neuritis were negative for both AQP-4 antibody and MOG antibody, labeling these patients as "double-negative" optic neuritis. Twelve percent of the patients were positive for AQP-4 antibodies, and 10% were positive for MOG antibodies. Only 4% of the double-negative group was diagnosed with MS. Noteworthy differences between the MOG and NMO groups include more isolated disease of the optic nerve with more severe ocular findings (disc edema and pain), better outcomes with immunotherapy, and less female preponderance in the MOG group. The AQP-4 antibody group had more disease spread to the spinal cord and more visual field deficits [14].

When vision loss is profound, sequentially bilateral, or simultaneously bilateral, it is more likely due to NMO or MOGAD [11,12]. The guidelines for the treatment of typical optic neuritis with IVMP have been established since 1992 [15,16]. However, NMO and MOGAD are often steroid-resistant with better responsiveness to PLEX therapy [17]. Classically, these conditions are either serological single positive or double positive for NMO autoantibodies and MOG autoantibodies. However, in the case of double-negative optic neuritis, which is negative for NMO autoantibodies and MOG autoantibodies, the literature on treatment responsiveness to PLEX is scarce. One retrospective study of 23 patients with steroid-unresponsive optic neuritis found that 48% of patients showed a significant improvement in visual acuity, with 30%-85% improvement in visual activity. In this study, steroid-unresponsiveness was defined as patients who received one-gram per day of IVMP at onset of symptoms for three to five days, and a second pulse two weeks later of two-grams per day for a similar three to five days without a 50% recovery of visual acuity in either eye [18].

We present a case of double-negative, sequential bilateral vision loss that responded favorably to PLEX. Following standard IVMP therapy, our patient’s symptoms actually worsened, and she subsequently developed bilateral vision loss. She had characteristic MRI findings in the optic nerves bilaterally. CT imaging of the chest, abdomen, and pelvis revealed no abnormal findings. Serology was negative for NMO autoantibodies, MOG autoantibodies, and CSF was negative for oligoclonal IgG bands. Additionally, further workup, including an anti-phospholipid antibody screen, antinuclear antibody (ANA), and rapid plasma reagin (RPR), were negative. After failing a second course of steroid therapy, the patient received PLEX therapy, and the visual recovery was remarkable. The rapid response to PLEX therapy in the setting of double-negative, steroid-resistant optic neuritis is quite unique. This case supports the growing evidence that despite NMO and MOG autoantibody negativity, initiation of PLEX therapy in steroid-resistant optic neuritis may be highly beneficial.

## Conclusions

Our case is a dramatic instance of treatment-resistant double-seronegative bilateral optic neuritis responsive to PLEX. Despite seronegativity, our case demonstrates that a suspected inflammatory optic neuritis may be treated empirically with PLEX with excellent results despite failure of standard treatment with IVMP. It is imperative to look for underlying ischemic or other auto-immune etiologies. By the same token, it is imperative, in order to salvage precious vision, not to shy away from invasive procedures such as PLEX when an inflammatory/autoimmune etiology is suspected and established therapeutic options fail.
